# The Emergence of *Fusarium oxysporum* f. sp. *apii* Race 4 and *Fusarium oxysporum* f. sp. *coriandrii* Highlights Major Obstacles Facing Agricultural Production in Coastal California in a Warming Climate: A Case Study

**DOI:** 10.3389/fpls.2022.921516

**Published:** 2022-06-13

**Authors:** Lynn Epstein, Sukhwinder Kaur, Peter M. Henry

**Affiliations:** ^1^Department of Plant Pathology, University of California, Davis, Davis, CA, United States; ^2^United States Department of Agriculture, Agricultural Research Service, Salinas, CA, United States

**Keywords:** *Apium graveolens* var. *dulce*, celery, cilantro, effectors, emerging pathogen, *Fusarium oxysporum* f. sp. *apii*, *Fusarium oxysporum* f. sp. *coriandrii*, *Fusarium oxysporum* species complex

## Abstract

Currently, *Fusarium oxysporum* f. sp. *apii* (*Foa*) race 4 in celery and *F. oxysporum* f. sp. *coriandrii* (*Foci*) in coriander have the characteristics of emerging infectious plant diseases in coastal southern California: the pathogens are spreading, yield losses can be severe, and there are currently no economical solutions for their control. Celery, and possibly coriander, production in these regions is are likely to have more severe disease from projected warmer conditions in the historically cool, coastal regions. Experimental evidence shows that *Foa* race 4 causes much higher disease severity when temperatures exceed 21°C. A phylogenomic analysis indicated that *Foa* race 4, an older, less virulent, and uncommon *Foa* race 3, and two *Foci* are closely related in their conserved genomes. These closely related genotypes are somatically compatible. *Foa* race 4 can also cause disease in coriander and the two organisms readily form “hetero” conidial anastomosis tubes (CAT), further increasing the likelihood of parasexual recombination and the generation of novel pathotypes. A horizontal chromosome transfer event likely accounts for the difference in host range between *Foci* versus *Foa* races 4 and 3 because they differ primarily in one or two accessory chromosomes. How *Foa* race 4 evolved its hyper-virulence is unknown. Although the accessory chromosomes of *Foa* races 3 and 4 are highly similar, there is no evidence that *Foa* race 4 evolved directly from race 3, and races 3 and 4 probably only have a common ancestor. *Foa* race 2, which is in a different clade within the *Fusarium oxysporum* species complex (FOSC) than the other *Foa*, did not contribute to the evolution of race 4, and does not form CATs with *Foa* race 4; consequently, while inter-isolate CAT formation is genetically less restrictive than somatic compatibility, it might be more restricted between FOSC clades than currently known. Other relatively new *F. oxysporum* in coastal California include *F. oxysporum* f. sp. *fragariae* on strawberry (*Fof*). Curiously, *Fof* “yellows-fragariae” isolates also have similar core genomes to *Foa* races 4 and 3 and *Foci*, perhaps suggesting that there may be core genome factors in this lineage that favor establishment in these soils.

## Introduction

*Fusarium oxysporum* f. sp. *apii* (*Foa*) race 4 is a pathogen of celery (*Apium graveolens* var. *dulce*) that causes an emerging disease in California, United States. *Foa* race 4 apparently arose in approximately 2013 (Epstein et al., 2017), is highly aggressive at temperatures above 22°C (Kaur et al., 2022), has been spreading in its geographic distribution within California, and currently cannot be controlled via either host resistance or economical methods that reduce the pathogen abundance in infested soil. Although *F. oxysporum* f. sp. *apii* have been long-recognized, first by Wollenweber in 1917, (Snyder and Hansen, 1940; Armstrong and Armstrong, 1981; Edel-Hermann and Lecomte, 2019), *Foa* are not currently as well known by the scientific community as *forma speciales* (ff. spp.) such as *lycopersici* on tomato, *cubense* on banana, *conglutinans* on Arabidopsis and cabbage, etc. (Husaini et al., 2018; Yang et al., 2020). Whole genome sequencing and analysis was recently conducted for *Foa* races 2, 3, and 4, and *F. oxysporum* f. sp. *coriandrii* (*Foci*), a pathogen of coriander (synonym, cilantro; *Coriandrum sativum;* Henry et al., 2020). In this review, we highlight aspects of the biology of *Foa* and its host–pathogen interactions that are potentially relevant for a broader understanding of other *Fusarium* pathosystems.

## History

**FIGURE 1:**
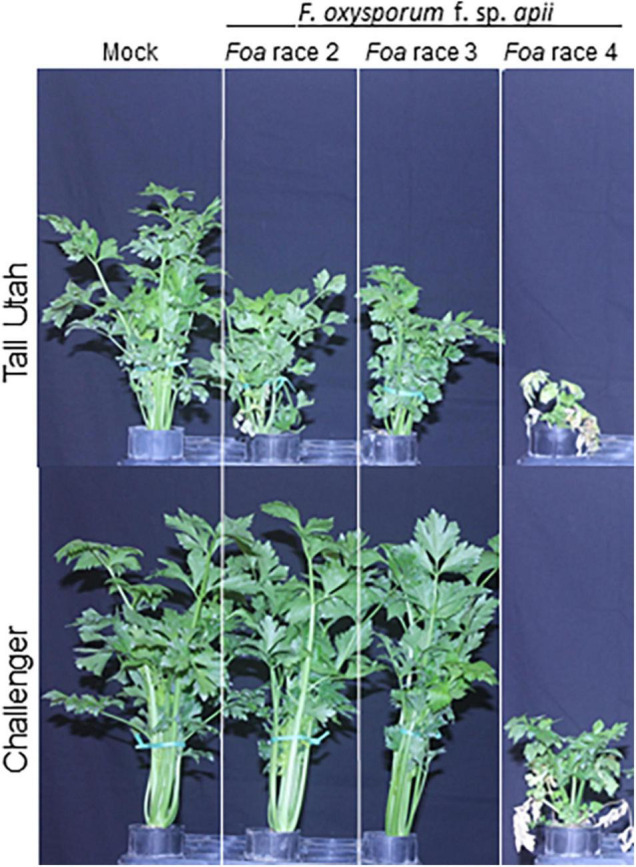
Virulence of three *F. oxysporum* f. sp. *apii* (*Foa*) races on celery cultivars Tall Utah and Challenger. Two-month-old celery cultivars were transplanted into uninfested soil (mock) or soil infested with a *Foa* race. After 49 days, the median plant (*n* = 20) in height was photographed. Tall Utah 5270R-Improved is susceptible to the three races and Challenger is resistant to *Foa* races 2 and 3 but susceptible to *Foa* race 4. The image is a portion of a figure from Henry et al. (2020). BMC Genomics 21, No. 730 and is reprinted in accordance with BMC Genomics.

What we now call *Foa* race 1, causal agent of Fusarium yellows of celery, is polymorphic (i.e., highly variable in their two-locus haplotypes), and apparently endemic in European and United States soils (Nelson et al., 1937; O’Donnell et al., 2009). In California, *Foa* race 1 strains are only virulent on heirloom, “yellow” celery (*Apium graveolens* var. *dulce*) cultivars that have not been commercially important since the 1950’s (Epstein et al., 2017). *Foa* race 2 was first observed in approximately 1959 on the “green” Pascal-type celery cultivars, e.g., Tall Utah, in California (Otto et al., 1976) (Figure 1); thereafter, race 2 was distributed, presumably on farm equipment, into all celery production areas in California. After *Foa* race 2 was established in California, it was apparently transported to other states (Subbarao and Elmer, 2002), Canada (Cerkauskas and Chiba, 1991), Japan (Akanuma and Shimizu, 1994), and Argentina (Lori et al., 2016), presumably on infested seed. If celery seed is produced in a field with *Foa* inoculum in the soil, a presumably low percentage of seed can be contaminated by wind-blown soil particles. Population sampling suggests that *Foa* race 2 is a monomorphic clonal lineage in the *Fusarium oxysporum* species complex (FOSC) clade 3 (Epstein et al., 2017). Largely based on somatic compatibility groups, *Foa* race 3 (Puhalla, 1984) was also present at least for some time in California, but is not highly virulent and has never been an important pathogen. More recently, *Foa* race 3 was identified in Costa Rica (Retana et al., 2018). After introgression of gene(s) for resistance from celeriac (*Apium graveolens* var. *rapaceum;* Orton et al., 1984b), *Foa* race 2 (and any race 3) were adequately controlled by resistant cultivars, e.g., cv. Challenger (Figure 1). *Foa* race 4 apparently first appeared in California in Camarillo, Ventura County in ca. 2013. Within a decade after its first sighting, race 4 had been moved, most likely on farm equipment, first to all celery production areas within Ventura county on the south coast, and more recently to Monterey county on the central coast. *Foa* race 1 haplotypes, *Foa* race 3, and *Foa* race 4 are in FOSC clade 2, which means that the newly emerged race 4 is only more distantly related in its core genome to *Foa* race 2.

*Foci* was considered an insufficiently documented *forma specialis* (Edel-Hermann and Lecomte, 2019), but our recent work should change that. The disease was first reported in California in Santa Barbara County (Koike and Gordon, 2005). In Ventura county, during the time period in which *Foa* race 4 apparently arose, there was also an increase in Fusarium wilt in coriander, caused by *Foci*; similar to *Foa* race 4, the *Foci* are highly virulent on their host. Interestingly, *Foa* race 4 can cause modest disease in coriander, although *Foci* are more virulent on coriander than *Foa* race 4; both pathogens can co-infect coriander (Henry et al., 2020). The *Foci* are avirulent on celery. Both celery and coriander are in the family Apiaceae, and the genomic similarities between *Foa* race 4 and *Foci* suggest there may be common susceptibility factors in celery and coriander.

## Symptoms

**FIGURE 2:**
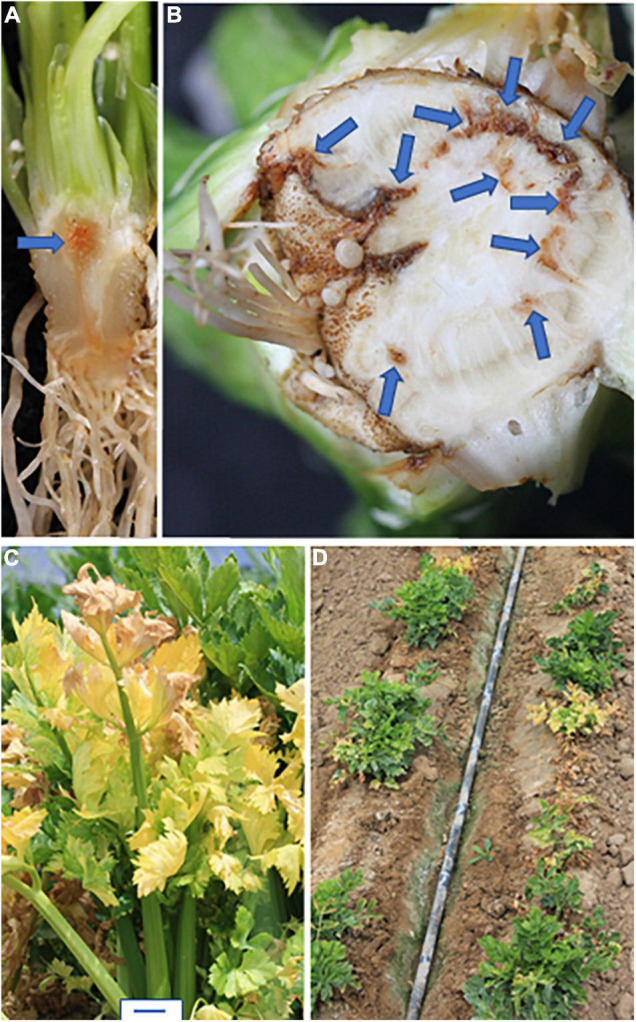
Symptoms of *F. oxysporum* f. sp. *apii* (*Foa*) in celery. **(A,B)** Vascular discoloration in the crown (shown here) and roots. These plants were infected with *Foa* race 4. **(A)** In young transplants, the discoloration is orangish-brown. **(B)** In older plants, the discoloration is darker brown. This field-grown celery plant had vascular discoloration on more than 25% of the circumference of the crown and was severely stunted. **(C)** Chlorosis of older leaves, here caused by *Foa* race 2. **(D)** Death and severe stunting of young plants caused by *Foa* race 4. Size bar = 2 cm.

All *Foa* induce celery to produce a vascular discoloration in infected roots and crown; in young transplants, the discoloration is orangish-brown (Figure 2A), and in older plants, the discoloration is a darker brown (Figure 2B). In severe cases, particularly with *Foa* race 4, the discoloration can extend into the base of the petioles. Stunting is strongly associated with vascular discoloration on more than 1/4 of the circumference of the vascular ring in the crown (Kaur et al., 2022). The extent of stunting and vascular discoloration is also associated with more *Foa* biomass in the celery crown (Kaur et al., 2022). Both *Foa* races 2 and 4 can cause some chlorosis, frequently in a lower leaf, but chlorosis (Figure 2C), is typically more noticeable in a field with race 2 than race 4. Both *Foa* races 2 and 4 cause stunting and a loss of yield and quality. However, at similar inoculum levels, particularly in soil temperatures above 21°C, *Foa* race 4 causes far more severe disease than *Foa* race 2 (Figure 1). In addition to severe stunting, *Foa* race 4 can cause death (Figure 2D), particularly on young plants; typically, the progression to death begins with stunting, sometimes with lower leaf chlorosis and proceeds to wilting of the entire plant and death. In the field, particularly in wet conditions, race 4 infections can result in root necrosis/sloughing off of the roots and/or a crown rot.

In contrast to celery, which is transplanted into the field in California, coriander is direct-seeded. As a consequence, although *Foci* produce similar symptoms on coriander as *Foa* race 4 produces on celery, *Foci*-infected coriander can also have poor seedling emergence. In the field, *Foci*-infected plants are stunted, often with chlorotic lower foliage (Koike and Gordon, 2005); the vascular discoloration in the roots and crown can extend into the petioles. Particularly when younger plants are infected, typical disease progression includes sudden wilting and rapid death. Fibrous roots are sloughed off and the taproot is often rotted. With *Foci* in coriander, the vascular discoloration is reddish to light brown.

## Lessons From *Fusarium oxysporum* f. sp. *apii* and *Fusarium oxysporum* f. sp. *coriandrii* in California

### Genetic Plasticity Challenges Disease Management and Molecular Diagnostics

#### Few Genetic Differences Can Cause Major Differences in Host Specificity and Virulence

In individual strains of *F. oxysporum*, chromosomes can be classified as part of either the “core” genome that is conserved amongst strains or the “accessory” (i.e., dispensable) genome that is variable amongst strains (Yang et al., 2020). A landmark discovery by Ma et al. (2010) demonstrated that accessory chromosomes could be horizontally transferred between strains of *F. oxysporum* with the genes required for pathogenicity in a specific host. Specifically, they demonstrated the transfer of a chromosome from a tomato pathogen (Fol4287) to a previously non-pathogenic recipient (Fo47); the transformed Fo47^+^ was pathogenic on tomato. Since this discovery, multiple pathogenicity chromosomes in the accessory genome, and in some cases, just portions of a pathogenicity chromosome, have been identified that encode for all the genes necessary to transform a non-pathogen into a pathogen of a specific crop, including cucumber, melon, and cabbage (Vlaardingerbroek et al., 2016; van Dam et al., 2017; Li et al., 2020; Ayukawa et al., 2021). The work has revealed that relatively few genetic differences can have profound impacts on pathogenicity and host range.

**FIGURE 3:**
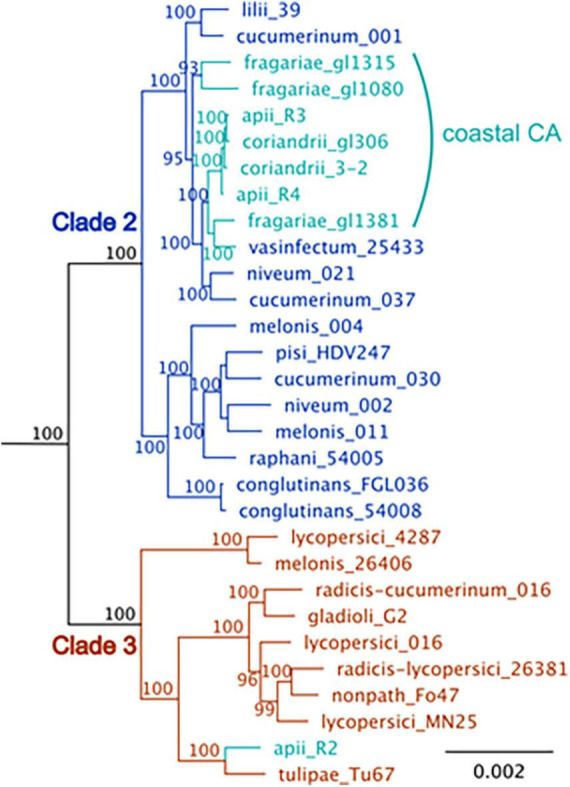
A maximum-likelihood phylogenetic tree of *F. oxysporum* isolates that cause disease in celery (apii), strawberry (fragariae), and coriander (coriandrii) in comparison to selected assemblies of *F. oxysporum* from NCBI. The figure is modified from Henry et al. (2020) with the addition of three *F. oxysporum* f. sp. *fragariae* sequences from Henry et al. (2021). BUSCO v. 2.0 (Benchmark of Unique Single Copy Orthologs) was used to identify 2,718 full-length, single-copy genes that were in all strains; these genes are primarily in the core genome. Sequences were aligned with MUSCLE and concatenated into a single ∼ 5.5 Mbp sequence. The tree was generated with RaxML with the general time-reversible evolutionary model, rooted with *F. oxysporum* f. sp. *cubense* tropical race 4, which is in *Fusarium oxysporum* species complex (FOSC) clade 1. Support for the tree is based on 1000 bootstrap replicates; bootstrap values below 70 are not shown. The coastal California isolates are shown in turquoise. *F. oxysporum* f. sp. *apii* race 2 (apii_R2), is in FOSC clade 3. The other *F. oxysporum* f. sp. *apii* (apii, R3, race 3) and (apii race 4, apii_R4) are in FOSC clade 2.

**FIGURE 4:**
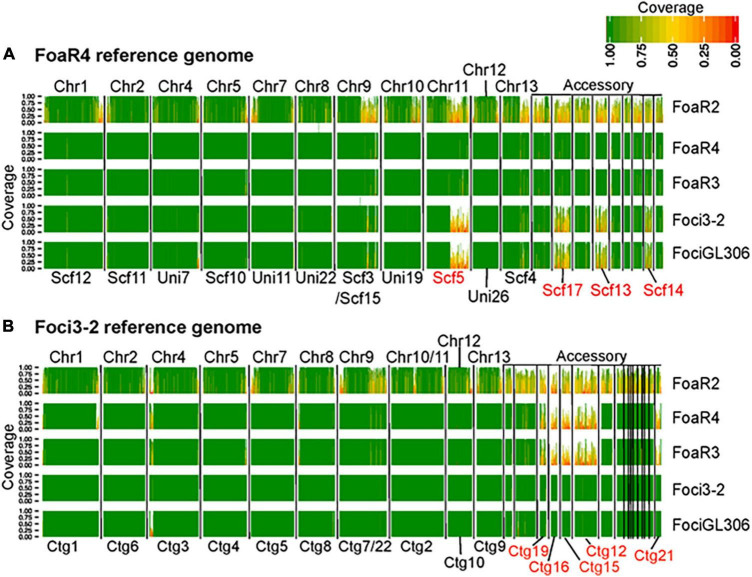
A comparative genomic analysis showing the extent of conservation in the core and accessory genomes between *F. oxysporum* f. sp. *apii* (*Foa*) race 4 and f. sp. *apii* races 2 and 3, and two strains of *F. oxsporum* f. sp. *coriandrii* (*Foci*). For this analysis, we used **(A)** *Foa* race 4 and **(B)** *Foci* 3–2 as reference assemblies, based on PacBio and Illumina sequencing. For each of the five strains, we mapped 6.5 Gbp (∼100× coverage) of quality-filtered Illumina reads onto each reference, and calculated the proportion of coverage of each 10 kbp window in the reference assemblies. Here, only contigs with a length greater than 150 kbp are shown and are separated by vertical black lines. The darkest green sections of the histogram have a 100% coverage and the reddest sections have coverage close to 0%. Coverage of 0.5 (yellow) indicates that only 5 kbp of the 10 kbp segment had Illumina coverage. The core chromosome (Chr) number of the *F. oxysporum* f. sp. *lycopersici* 4287 reference are labeled above each plot toward the left. The accessory portion of the reference genomes is indicated on the upper right of each figure. The IDs for contigs (Ctg) and scaffolds (Scf) that are associated with host specificity in our two references are lettered in red, below the plots. Each row shows the coverage from a single isolate, which is noted to the right of the graph (*Foa* race 2, *Foa*R2; *Foa* race 3, *Foa*R3). The image is a figure from Henry et al. (2020) (BMC Genomics 21, No. 730) and is reprinted in accordance with BMC Genomics.

Although there are profound differences in symptom severity and host range between *Foa* race 3, race 4, and *Foci*, they have similar genomes. In a comparison of 2,718 conserved, single copy orthologs in the core genome, we discovered that the low-virulence *Foa* race 3, highly virulent *Foa* race 4, and *Foci* isolates are tightly clustered in a monophyletic group (Figure 3; Henry et al., 2020). Given this close phylogenetic relationship, many genomic similarities are vertically inherited and identical by descent in these three groups. Figure 4 shows an analysis of the extent of conservation and lack thereof of both the core and accessory genomes between the three *Foa* and two *Foci* strains. *Foa* race 3 and race 4 are nearly identical in their core and their accessory genomes (Figure 4A), despite their profound differences in virulence (Henry et al., 2020). The two *Foci*, which were collected 12 years apart from two counties, have highly similar core and accessory genomes to each other (Figure 4B; Henry et al., 2020). In contrast, the two *Foci* differ from *Foa* race 3 and race 4 in approximately 1/3 of their accessory genome (Henry et al., 2020), i.e., probably in one or possibly two accessory chromosomes. The differences in host range between these *Foa* and *Foci* strains are likely to be caused by presence/absence variation in the divergent portion of the accessory genome.

The similarity between the accessory genomes of *Foa* race 4 and race 3 suggests that few genetic differences account for the hypervirulence in race 4. However, perhaps as because as small a difference as a single nucleotide polymorphism might account for the hyper-virulence, to date, we have not succeeded in generating a viable hypothesis of why *Foa* race 4 is hyper-virulent. None of the highly expressed putative effectors in *Foa* race 4, which would be prime candidates for disruption, are absent in *Foa* race 3. Furthermore, *Foa* race 3 produces comparatively little biomass *in planta*, making it extremely difficult to obtain sufficient RNAseq reads for *Foa* race 3 infection-specific transcriptome profiling (Kaur and Epstein, unpublished). We initially identified one potential avirulence effector in *Foa* race 3 in its accessory genome (Kaur, Epstein, Henry, and Stergiopolos, unpublished): the DNA (GenBank MT349842.1) is absent in *Foa* race 4, and based on reverse transcriptase real-time PCR, the gene is expressed *in planta* in race 3-infected celery but not in race 3 *in vitro*, or negative controls. However, after performing an *Agrobacterium tumefaciens*-mediated knock-out (Gold et al., 2017), the race 3 knock-outs have the same virulence as the wild type and ectopic recombinant controls, indicating that this gene is not an avirulence factor (data not shown).

As part of an investigation of potential effectors and virulence factors in *Foa* race 4, we quantified gene expression *in planta* within the crown compared to in culture. Two of the notable genes that were highly expressed and up-regulated *in planta* were transposons (Henry et al., 2020). Clearly, transposon activity in *F. oxysporum* contributes to isolate diversity, the potential for the evolution of new pathotypes, and potentially the loss of the informative markers used in molecular diagnostics.

#### Horizontal Chromosome Transfer Could Account for the Major Difference in the Accessory Genomes of *Fusarium oxysporum* f. sp. *apii* Race 4 and *Fusarium oxysporum* f. sp. *coriandrii* and for Their Differences in Host Range, but Not the Extreme Differences in Virulence Between *Fusarium oxysporum* f. sp. *apii* Races 3 and 4

Our work further supports the view that accessory chromosomes are present in a “mosaic” pattern between strains, where a range of presence/absence variation among similar chromosomes can produce new host specificities (Figure 4). Although our assemblies are fragmented in 50–75 contigs per genome, we can deduce that horizontal chromosome transfer must have occurred from an unknown source to either the *Foci* or to the progenitor of the *Foa* FOSC clade 2. This is the most parsimonious explanation for the presence/absence variation in approximately 1/3 of the accessory genome between these isolates that otherwise have nearly identical, vertically inherited genomes. In this scenario, the gain of one or more accessory chromosomes would have been accompanied with a concomitant loss of another accessory chromosome(s). This further substantiates the potential for these organisms and for a broader array of *F. oxysporum* to generate new combinations of accessory chromosomes that will contribute to its ability to evolve new pathogenic genotypes in the future.

In contrast, as illustrated in Figure 4, the hypervirulence of *Foa* race 4, in comparison to *Foa* race 3, was not caused by the acquisition of a large (> 0.5 Mbp) accessory chromosomal region from either any of the strains that we sequenced or from any other full genome-sequenced *F. oxysporum* (Henry et al., 2020). The *Foa* race 4 strain shares almost all (> 95%) of its accessory genome with *Foa* race 3.

#### Conidial Anastomosis Tube Formation and Somatic Compatibility Between *Fusarium oxysporum* f. sp. *apii* Race 4 and *Fusarium oxysporum* f. sp. *coriandrii* Increase the Potential for New Recombinants to Emerge

In general, somatic compatibility groups (SCGs), also known as vegetative compatibility groups or VCGs, have been a biologically useful way of indicating whether two *F. oxysporum* are closely related (Correll et al., 1986a). After our initial phylogenetic data indicated that an older *Foa* race 3, which we retrieved from a culture collection, and the emergent *Foa* race 4 were indistinguishable in our initial phylogenetic data (Epstein et al., 2017), we determined that *Foa* race 3 and 4 are in the same SCG, which indicates that they are closely related and share the requisite somatic compatibility gene alleles. Similarly, after we discovered the similarity in DNA sequence between *Foci* and *Foa* races 3 and 4, we demonstrated that *Foci* are also in the same SCG as *Foa* race 3 and race 4 (Henry et al., 2020). We are not aware of another example where organisms in two different *formae speciales* are in the same SCG. This somatic compatibility means that horizontal chromosome transfer could readily occur *via* hyphal fusion between co-localized *Foci* and *Foa* races 4 or 3. These strains can occur in the same fields and could be brought into close proximity by co-infection of root tissues.

**FIGURE 5:**
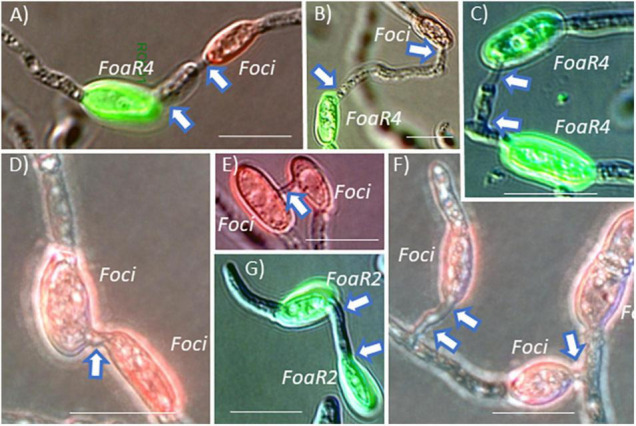
*F. oxysporum* microconidia were pre-stained with a fluorescent dye conjugate and then mixed with a differently labeled strain. After incubation on polystyrene in conditions conducive for conidial anastomosis tube (CAT) formation, “homo” and “hetero” CAT formation was enumerated (as shown in the original publication). **(A,B)** *F. oxysporum* f. sp. *apii* race 4 (*Foa*R4) and *F. oxysporum* f. sp. *coriandrii* 3-2 (*Foci*) formed hetero-CATs as frequently as each strain formed homo-CATs **(C,D)**. **(E)** In compatible interactions, CATs were most commonly formed between two microconidia, but occasionally, CAT clusters with three microconidia **(F)** were observed. *F. oxysporum* f. sp. *apii* race 2 (*Foa*R2) also formed homo-CATs **(G)** but *Foa*R2 and *Foa*R4 were never observed to form a hetero-CAT. Short CATs are indicated with a single arrow and longer CATs are indicated with an arrow at each end of the CAT. Size bars = 10 μm. The image is a portion of a figure from Henry et al. (2020). (BMC Genomics 21, No. 730) and is reprinted in accordance with BMC Genomics.

Conidial anastomosis tubes are also an avenue for a horizontal chromosome transfer of an accessory/pathogenicity chromosome because nuclei can migrate across a CAT (Kurian et al., 2018), and importantly, CAT formation is a less restrictive barrier for nuclear entry than somatic/hyphal compatibility in *F. oxysporum* (Shahi et al., 2016), *Colletotrichum lindemuthianum* (Ishikawa et al., 2012), and *Verticillium dahlia*e (Vangalis et al., 2021). Microconidia are frequently produced *in planta* in the xylem to facilitate systemic plant colonization. These conidia presumably could undergo CAT fusion. Because *Foa* race 4 and *Foci* can co-infect coriander and can form *Foa* race 4-*Foci* hetero-CATs (Figures 5A,B), coriander and, perhaps, celery may be a likely location for accessory chromosome transfer and evolution of new genotypes. We note that *Foa* race 4 and *Foci* form hetero-CATs (Figures 5C–F) as frequently as they form “homo” CATs (Henry et al., 2020). Thus, if *Foa* race 4 and *Foci* co-occur in conducive conditions for either somatic compatibility or CAT formation, they could undergo nuclear transfer and then horizontal chromosome transfer; such transfer could result in the evolution of a new genotype that could evade host recognition, increase virulence, and confound molecular detection of the new strain.

#### Close Phylogenomic Relationships and the Potential for Recombination Complicate Efforts to Develop Molecular Diagnostics Specific to *Fusarium oxysporum* f. sp. *apii* Race 4 and *Fusarium oxysporum* f. sp. *coriandrii*

Using our whole genome shotgun sequences of *Foa* races 2, 3, and 4, and *Foci*, along with the NCBI GenBank wgs and selected SRA databases, we were able to design and validate PCR primers for detection of *Foa* races 2 and 4 and *Foci* either directly from plants or from culture (Henry et al., 2020). While empirical testing of a strain collection and *in silico* testing revealed comparatively few problems, not surprisingly, it is difficult to develop primers with no false positives. For example, in an *in silico* analysis of 437 *F. oxysporum* genomes in the Genbank wgs, and an additional 329 other *Fusarium* spp., the *Foa* race 4 primers FOAR4-447 would amplify 1.8% of the non-target *F. oxysporum* and one *F. secorum*.

Our preliminary results further suggest that recombination is occurring between strains of *Foa* race 4 and *Foci* in agricultural fields. After isolating from symptomatic coriander, we assembled a small collection of seven isolates that were *bona fide Foci* in that each caused disease on coriander but not on celery (Henry et al., 2020). In contrast to the other six *Foci* isolates, *Foci*10T was PCR-negative for two PCR primers on the host-specific *Foci* accessory genome (FOCI2-21 and FOCI-g_c31), and was PCR-positive for *Foa* race 4 primer R4-447, which is located an accessory region not shared by other, whole genome sequenced *Foci* isolates (Henry et al., 2020). This suggests that *Foci*10T lost regions of accessory chromosomes that are common to *Foci* and gained an *Foa* race 4-specific accessory chromosome region while retaining the avirulence-on-celery phenotype. This observation highlights the strong potential for recombination to confound efforts to distinguish these *formae speciales* solely with molecular markers.

### There May Be a Barrier for Genetic Exchange Between *Fusarium oxysporum* f. sp. *apii* Race 4 and Race 2, and Consequently Between Other Members of *Fusarium oxysporum* Species Complex Clades 2 and 3

*Fusarium oxysporum* f. sp. *apii* (*Foa)* race 4 and *Foa* race 2 can be isolated from the same celery plant. When we initially observed co-occurring *Foa* races 2 and 4, and knew that the possibly extinct *Foa* race 3 was closely related to the new and highly aggressive *Foa* race 4, we postulated that *Foa* race 4 was perhaps the result of the acquisition of a *Foa* race 2 accessory chromosome in a *Foa* race 3 background. However, as illustrated in Figure 4A, the phylogenomic analyses indicate that *Foa* race 4 did not evolve from *Foa* race 2 and did not receive a portion of a chromosome from this extant pathogen of celery. Moreover, in our enumeration of CATs in assays with *Foa* race 2 and race 4 microconidia, we observed race 4-race 4 homo-CATs (Figure 5C) and race 2-race 2 homo-CATs (Figure 5G), but never a race 2-race 4 hetero-CAT. While one might argue that we would not have detected very low frequency events, nothing that we ever observed suggested that a *Foa* race 2 and a *Foa* race 4 might even start the process of CAT formation. Consequently, although CAT formation is a less restrictive conduit for nuclear entry than somatic/hyphal compatibility (Shahi et al., 2016), we postulate that there are more barriers than currently appreciated for inter-isolate CAT formation within the FOSC, and that barriers to CAT formation between FOSC clades may restrict nuclear entry and consequently accessory chromosome transfer. We note that the study on *F. oxysporum* CATs (Shahi et al., 2016) demonstrated transfer between two strains that are in FOSC clade 3: *F. oxysporum* f. sp. *lycopersici* 4287 and the biocontrol strain Fo47. Experimentally, using tagged pathogenicity chromosomes, Rep and colleagues have demonstrated that horizontal chromosome transfer can transform a non-pathogenic isolate (Fo47) into a pathogen of tomato (Ma et al., 2010), multiple cucurbits (van Dam et al., 2017), and melons (Li et al., 2020). However, those demonstrations, [with Fol4287, Forc016, and Fom001 (synonym, NRRL26406) and Fom005, respectively] are all within FOSC clade 3 (Figure 3), and the transfer between FOSC clades 2 and 3 has not been reported (van Dam et al., 2016; Henry et al., 2020). A critical question for future research in the broader Fusarium community is if two strains co-occur as microconidia within a host, are there limits to CAT formation between FOSC clades?

### Climate Change Will Likely Exacerbate Fusarium Wilt Disease Severity and Incidence in Coastal California

#### The Disease Severity of *Fusarium oxysporum* f. sp. *apii* Race 4 in Celery and the Growth of *Fusarium oxysporum* f. sp. *apii* Race 4 *in vivo* Is Dramatically Increased as Temperatures Exceed 22°C

A major difference between *Foa* race 2 and *Foa* race 4 is that while both *Foa* race 2 and *Foa* race 4 are temperature-sensitive, the magnitude of the high temperature response in *Foa* race 4 is significantly greater (Kaur et al., 2022). In the celery cultivar Sonora, which is susceptible to both *Foa* race 2 and race 4, at 18°C, there were no significant (α = 0.05) differences between races in either stunting, i.e., the reduction in plant height, or in the concentration of the *Foa* in the crown, as estimated by qPCR. In contrast, at temperatures of 22, 24, and 26°C, there was significantly (α = 0.05) more *Foa* race 4 than *Foa* race 2 DNA *in planta* in crowns. Consequently, the predicted global warming in California (Oakely et al., 2019) will increase the *Foa* race 4 disease threat.

Global warming in California (Oakely et al., 2019) could have a large impact on the growth of multiple FOSC pathogens and consequently the production of high-quality crops in California. The FOSC typically have temperature optima from 25 to 28°C (Kaur et al., 2022). There are multiple examples of diseases caused by *F. oxysporum formae speciales* in which disease severity in susceptible varieties increases as temperatures increase from 22 to 28°C (e.g., f. sp. *ciceris* in chickpea, *cepae* in onions, *conglutinans* in cole crops, *lactucae* in lettuce, *lycopersici* in tomatoes, *medicaginis* in alfalfa, and *melonis* in melons; Bosland et al., 1988; Navas-Cortés et al., 2007; Scott et al., 2010; Jelínek et al., 2019). In the case of the diseases caused by f. sp. *conglutinans* on crucifers and *ciceris* on chickpeas, higher temperatures also decreased expression of resistance (Bosland et al., 1988; Landa et al., 2006). We note that celery, a cool weather crop, has a temperature optimum of 16 to 18°C to perhaps 21°C (Rubatzky et al., 1999; Smith, 2021), and that many of the crops in California that have an important *F. oxysporum* pathogen are also cool weather crops, including strawberry, chickpea, onions, cabbage, lettuce, alfalfa, spinach, caneberries, and garlic.

#### *Fusarium oxysporum* f. sp. *apii* and *Fusarium oxysporum* f. sp. *coriandrii* Are Only Two of the *Fusarium oxysporum* That Have Become More Important in Coastal California, United States Agriculture in the 21^st^ Century

Based on disease reports, *Foa* races 2 and 4 appear to have originated in California fields. There are several other crops that are often grown in the same locales as celery and coriander in California that are infected by other *F. oxysporum* ff. spp. Here, we primarily mention pathogenic *F. oxysporum* on lettuce and strawberry. It is important to remember that *F. oxysporum* typically survive for many years in the absence of a plant host because although the host ranges for diseases are generally very narrow, *F. oxysporum* commonly infect and reproduce to some extent in root cortical cells of non-hosts (Henry et al., 2019).

*Fusarium oxysporum* f. sp. *lactucae* apparently originated in Japan and was then introduced into California presumably on contaminated lettuce seed in approximately 1990 (Paugh and Gordon, 2020). A single clonal lineage of f. sp. *lactucae* race 1 has now been transported, presumably on farm equipment, to all lettuce-growing areas in California, where the disease is problematic, particularly at higher temperatures (Scott et al., 2010; Paugh and Gordon, 2019). Based on SCGs, there has been some differentiation of isolates within the race 1 lineage (Paugh and Gordon, 2020). However, too few informative loci of f. sp. *lactucae* isolates have been sequenced to place the isolates in a robust phylogenetic tree.

*Fusarium oxysporum* f. sp. *fragariae* (*Fof*), a strawberry pathogen, was first reported in eastern Australia in 1962 and then in Japan in 1969, South Korea in 1974, and in multiple locales in the early 2000s including in California in 2006 (Henry et al., 2017). In California, the emergence of the chlorosis-inducing “yellows-*fragariae*” pathotype of *Fof* coincided with the phase-out of the soil fumigant methyl bromide, which essentially began in 1999 (Epstein, 2014). Currently, *Fof* is considered to be one of the three most important pathogens of strawberry in California. In contrast to *Foa* and f. sp. *lactucae* in California, which are in fewer clonal lineages, based on the conserved genome, *Fof* in California are variable, but fit into three distinct clonal lineages, of which all are in one subclade of FOSC clade 2 (Henry et al., 2021). Surprisingly, as we show in Figure 3, the three *Fof* from California are closely related to *Foa* races 3 and 4 and the *Foci* in their core genomes.

A phylogenomic analysis of an international collection of *Fof* (Henry et al., 2021) indicated that horizontal chromosome transfer of a pathogenicity/accessory chromosome was critical for *Fof* diversification of this *forma specialis*. Overall, in terms of core genome types and the pathogenicity chromosome, there are California *Fof* isolates in three groups: a group that includes isolates from western Australia that have a Y1 core and a T1080 accessory chromosome; a group that includes isolates from Japan with a Y2 core and a T1 accessory chromosome; and a group that includes isolates from Japan with a Y3 core and a T1381 accessory chromosome. Based on the international isolate collection, horizontal chromosome transfer of a pathogenicity chromosome has occurred at least four times. Thus, while the epiphytotic-causing strains of *Foa* and *Foci* may have originated in California, the problematic f. sp. *lactucae* and *fragariae* were introduced into California presumably on seed and on vegetative material, respectively (Okamoto et al., 1970; Nam et al., 2011; Pastrana et al., 2019). In the case of *Fof*, movement of the pathogen within California can occur on nursery-infected plants and/or on farm equipment.

#### As in Many Domesticated Crops, Celery Has a Narrow Genetic Base, and There Is a Need for a Broader Germplasm Collection With Potential Resistance Genes

Domesticated crops often have lost disease resistance genes that are present in wild progenitors (van de Wouw et al., 2010; Purugganan, 2019). The celery cultivar Ventura, which is susceptible to *Foa* races 2 and 4 (Kaur and Epstein, unpublished), has only 62 nucleotide binding site (NBS) disease resistance-type genes, all with leucine rich regions, including 10 with Toll/interleukin-1 receptors (TNL), 44 with coiled coil domains (CNL), and 8 with resistance to powdery mildew 8 (RPM8) (RNL) subtypes (Song et al., 2021).

Celery (*A. graveolens* var. *dulce*) can be crossed either with other celery, with celeriac (*A. graveolens* var. *rapaceum*), with “cutting celery” (*A. graveolens* var. *secalinum*), or with wild celery (*A. graveolens* var. *graveolens*). In the 1980s, UC Davis researchers introgressed resistance gene(s) to *Foa* race 2 (Orton et al., 1984a) from celeriac into celery, which ultimately resulted in the release of *Foa* race 2-resistant cultivars (Daugovish et al., 2008) such as Challenger and Sabroso. However, multiple celeriac accessions are resistant to *Foa* race 2 (Kaur and Epstein, unpublished). In contrast, very few *A. graveolens* accessions in the USDA and UC Davis *Apium* germplasm collections have resistance to *Foa* race 4 (Kaur and Epstein, unpublished). This reinforces the need for wild germplasm collections (Frese et al., 2018), particularly with wild *Apium graveolens* var. *graveolens*, which presumably have more resistance genes. Breeding programs would also benefit from wild germplasm that is adapted to warmer temperatures, i.e., above 21°C.

### Interactions With and Relationship to Other Strains of *Fusarium oxysporum*

#### Celery Is an Excellent Host for Studying the Interaction of Endophytes and Saprophytic Strains With a *Fusarium oxysporum* f. sp. *apii* Pathogen

Endophytic *F. oxysporum* are readily isolated from internal tissue from celery crowns, from both healthy plants and from the margins of symptomatic tissue in infected plants (Epstein et al., 2017). Indeed, celery plants infected with a pathogenic race frequently contain at least one non-pathogenic *F. oxysporum* strain (Schneider, 1984; Correll et al., 1986b; Epstein et al., 2017). We assembled a collection of 174 *F. oxysporum* isolates from celery with symptoms of a *Foa* infection, primarily, so that we would be able to determine whether the pathogen population in California, and particularly *Foa* race 2, had changed between 1993 and 2014 (Epstein et al., 2017). Almost half (48%) of the isolates were non-pathogenic. Twenty-three of the non-pathogenic isolates were in the *Foa* race 2 elongation factor 1-α/intergenic spacer (EF-1α/IGS) two-locus sequence haplogroup and two were in one of two *Foa* race 1 haplogroups. Therefore, ∼30% of the 83 non-pathogenic strains may have a core genome that is compatible with pathogenicity in celery and some of those isolates may have lost their pathogenicity in storage. The remaining 58 non-pathogenic isolates represented 20 phylogenetically diverse two-locus haplotypes, with 18 lineages nested within FOSC clade 3 and two within clade 2. Thirteen of the putatively non-pathogenic lineages were represented by singletons. We selected seven non-pathogenic isolates, with six from within FOSC clade 3, and obtained 250 bp paired-end Illumina sequence to at least 10X coverage and examined SNPs and DNA sequence of 10 phylogenetically informative genes from the non-pathogenic isolates, nine pathogenic isolates from celery, and twelve *F. oxysporum* reference genomes. The results confirmed that in contrast to the highly clonal *Foa* race 2, the non-pathogenic isolates were diverse, but clustered with the FOSC clade 3 subclade with the *F. oxysporum* biocontrol Fo47 (synonym, NRRL 54002). In an earlier study of non-pathogenic *F. oxysporum* from apparently healthy celery in California (Correll et al., 1986b), 28 non-pathogenic isolates were shown to be in 14 SCGs. Using these 14 SCG as testers, only 27% of the remaining 82 isolates in their collection were in one of these SCGs. That is, even healthy celery is a host of a highly diverse population of *F. oxysporum*. We note that we have not observed a role of selected non-pathogenic *F. oxysporum* in disease caused by *Foa* race 4 (Kaur and Epstein, unpublished).

#### Some Recent Name Changes in *Fusarium* Will Create Chaos for the Fusarium Community

Because of the importance of *Fusarium* spp. in agricultural production and in disease of many hosts, the Fusarium community needs to be able to communicate essential information on a range of topics from the population genetics to the results of diagnostic assays to a diverse audience, including other scientists, plant pathologists, pest control advisors, growers, regulators, medical practitioners, and the public. As a consequence, the Fusarium community (Geiser et al., 2013, 2021; O’Donnell et al., 2020) has resolved that both the phylogeny and the collective knowledge and needs of the Fusarium community and the public should be considered before making taxonomic revisions. If changes, such as the division of *F. oxysporum* into 16 species (Lombard et al., 2019) were accepted, it’s unclear what all the organisms in this review should be called, our text would have a needless layer of confusing complexity and perhaps ambiguity, and no one would have a better understanding about either the biology or control of these organisms. While the polyphyletic origin of *Foa* and other *forma speciales* is admittedly taxonomically problematic, the *ff. spp.* designations encode much useful information (Edel-Hermann and Lecomte, 2019). As a community, let’s strive to only adopt taxonomic changes that are biologically informative and improve our ability to communicate about all of the incredibly diverse strains in *F. oxysporum*, and the still insufficiently defined potential that they do and do not have to transmit their genes.

## Discussion and Conclusion

In 2021, 777 thousand tonnes of celery were produced in California,^1^ primarily in the south and central coasts (Daugovish et al, 2008). If the soil is infested with *Foa* race 4, the production of both celery, and to a lesser extent coriander, are threatened, particularly if the temperature exceeds 21°C. As with most *F. oxysporum*, *Foa* survive in the soil for many years. Full genome sequencing allowed the development of diagnostic PCR primers/probes that can be used to identify *Foa* races 2 and 4 and *Foci in planta* (Henry et al, 2020). Genome assembly of the *Foa* races and the race 4 relative, *Foci*, have allowed a view of both the core and accessory genomes of these pathogens; even though *Foa* race 2 and race 4 can co-infect celery, both the phylogenomics and experimental evidence indicate that horizontal chromosome transfer does not occur. In contrast, horizontal chromosome transfer could occur between *Foa* race 4 and *Foci* via either hypha or CATs, with the resultant formation of potentially new pathogen genotypes.

Within the last one to three decades, two other diseases caused by *F. oxysporum* have emerged in Coastal California agriculture: Fusarium wilt on lettuce, which is also more severe in warmer temperatures, and Fusarium wilt on strawberry. In contrast to *Foa* race 4 and *Foci*, which may be endemic, both f. sp. *lactucae* and f. sp. *fragariae* appear to have been introduced. Surprisingly, *Fof* is comparatively closely related to *Foa* race 4 and *Foci* (Figure 3); we do not know what this means in terms of the natural history of these organisms, but it seems unlikely that it’s a coincidence. We also do not know if *Foa* race 4 and *Fof* co-occur in conditions in which CATs form, but we do know that while *Foa* race 4 is not a strawberry pathogen, it can infect and survive in strawberry roots (Kaur and Epstein, unpublished). Regardless, the confluence of multiple factors indicate that coastal California will continue to be a hot spot for *F. oxysporum* diseases in the future: (1) *F. oxysporum* have an optimum of 25 to 28°C, the coastal climate is warming, the region will have more days in this near-optimum temperature range (Oakely et al., 2019), many of the intensive crops in this region, e.g., caneberries, celery, onions, cabbage, lettuce, spinach, and garlic, are better adapted for cooler temperatures, and disease in multiple *ff. spp.* is more severe at warmer temperatures; (2) the intensive agriculture, sometimes without crop rotation, allows production of high inoculum concentrations in the soil; (3) *F. oxysporum* inoculum is maintained in infested fields in multiple ways including, the pathogen can reproduce in non-host roots and debris at sufficient levels that populations persist despite rotation, and there is little knowledge of how to best manage crop residues so as to reduce inoculum concentration in soil; (4) *F. oxysporum* pathogens are routinely introduced into fields in soil and infected debris on harvesters and other equipment within and between counties, and on infected or infested seed and vegetative material that is part of global trade; (5) new pathotypes can arise via either CAT formation and horizontal chromosome transfer, or transposon activity, etc.; and (6) there are insufficient social factors that encourage growers to work toward long term disease control and multiple social factors that discourage it, including the economic pressure to farm for short-term profits and farming on leased land.

## Author Contributions

LE wrote the manuscript with input from PMH and SK. All authors conducted the research and approved the manuscript.

## Conflict of Interest

The authors declare that the research was conducted in the absence of any commercial or financial relationships that could be construed as a potential conflict of interest.

## Publisher’s Note

All claims expressed in this article are solely those of the authors and do not necessarily represent those of their affiliated organizations, or those of the publisher, the editors and the reviewers. Any product that may be evaluated in this article, or claim that may be made by its manufacturer, is not guaranteed or endorsed by the publisher.

## Footnotes

1
https://quickstats.nass.usda.gov/

## References

[B1] Akanuma R. Shimizu T. (1994). Epidemiology and control of Fusarium yellows of celery [*Apium graveolens*], 1: etiology of fusarium yellows of celery and occurrence of *Fusarium oxysporum* f. sp. apii race 2 in Nagano. *Bullet. Nagano Veg. Ornam. Crops Exp. Stat.* 8, 65–70.

[B2] Armstrong G. M. Armstrong J. K. (1981). “Formae speciales and races of *Fusarium oxysporum* causing wilt diseases,” in *Fusarium: Disease, Biology, and Taxonomy*, eds Nelson P. E. Toussoun T. A. James Cook R. (Pennsylvania: Pennsylvania State University Press), 391–399.

[B3] Ayukawa Y. Asai S. Gan P. Tsushima A. Ichihashi Y. Shibata A. (2021). A pair of effectors encoded on a conditionally dispensable chromosome of *Fusarium oxysporum* suppress host-specific immunity. *Commun. Biol.* 4:707. 10.1038/s42003-021-02245-4 34108627 PMC8190069

[B4] Bosland P. W. Williams P. H. Morrison P. H. (1988). Influence of soil temperature on the expression of yellows and wilt of crucifers by *Fusarium oxysporum*. *Plant Dis.* 72 777–780.

[B5] Cerkauskas R. F. Chiba M. (1991). Soil densities of *Fusarium oxysporum* f. sp. apii race 2 in Ontario, and the association between celery cultivar resistance and photocarcinogenic furocoumarins. *Can. J. Plant Pathol.* 13 305–314.

[B6] Correll J. C. Puhalla J. E. Schneider R. W. (1986a). Identification of *Fusarium oxysporum* f. sp. apii on the basis of colony size, virulence, and vegetative compatibility. *Phytopathology* 76 396–400.

[B7] Correll J. C. Puhalla J. E. Schneider R. W. (1986b). Vegetative compatibility groups among nonpathogenic root-colonizing strains of *Fusarium oxysporum*. *Can. J. Bot.* 64 2358–2361. 10.1139/b86-310

[B8] Daugovish O. Smith R. Cahn M. Koike S. Smith H. Aguiar J. (2008). *Celery Production in California.* Davis, CA: University of California, Agriculture and Natural Resources. 10.3733/ucanr.7220

[B9] Edel-Hermann V. Lecomte C. (2019). Current status of *Fusarium oxysporum* formae speciales and races. *Phytopathology* 109 512–530. 10.1094/PHYTO-08-18-0320-RVW 30461350

[B10] Epstein L. (2014). Fifty years since silent spring. *Annu. Rev. Phytopathol.* 52 377–402. 10.1146/annurev-phyto-102313-045900 25001457

[B11] Epstein L. Kaur S. Chang P. L. Carrasquilla-Garcia N. Lyu G. Cook D. R. (2017). Races of the celery pathogen *Fusarium oxysporum* f. sp. apii are polyphyletic. *Phytopathology* 107 463–473. 10.1094/PHYTO-04-16-0174-R 27938244

[B12] Frese L. Bönisch M. Nachtigall M. Schirmak U. (2018). Patterns of genetic diversity and implications for in situ conservation of wild celery (*Apium Graveolens* L. ssp. *graveolens*). *Agriculture* 8:129. 10.3390/agriculture8090129

[B13] Geiser D. M. Al-Hatmi A. M. S. Aoki T. Arie T. Balmas V. Barnes I. (2021). Phylogenomic analysis of a 55.1-kb 19-gene dataset resolves a monophyletic *Fusarium* that includes the *Fusarium Solani* species complex. *Phytopathology* 111 1064–1079. 10.1094/PHYTO-08-20-0330-LE 33200960

[B14] Geiser D. M. Aoki T. Bacon C. W. Baker S. E. Bhattacharyya M. K. Brandt M. E. (2013). One fungus, one name: defining the genus *Fusarium* in a scientifically robust way that preserves longstanding use. *Phytopathology* 103 400–408. 10.1094/PHYTO-07-12-0150-LE 23379853

[B15] Gold S. E. Paz Z. García-Pedrajas M. D. Glenn A. E. (2017). Rapid deletion production in fungi via agrobacterium mediated transformation of OSCAR seletion xonstructs. *J. Vis. Exp.* 12:55239. 10.3791/55239 28654073 PMC5608391

[B16] Henry P. Kaur S. Pham Q. A. T. Barakat R. Brinker S. Haensel H. (2020). Genomic differences between the new *Fusarium oxysporum* f. sp. apii (Foa) race 4 on celery, the less virulent Foa races 2 and 3, and the avirulent on celery f. sp. coriandrii. *BMC Genomics* 21:730. 10.1186/s12864-020-07141-5 33081696 PMC7576743

[B17] Henry P. M. Kirkpatrick S. C. Islas C. M. Pastrana A. M. Yoshisato J. A. Koike S. T. (2017). The population of *Fusarium oxysporum* f. sp. *fragariae*, cause of *Fusarium* wilt of strawberry, in California. *Plant Dis.* 101 550–556. 10.1094/PDIS-07-16-1058-RE 30677354

[B18] Henry P. M. Pastrana A. M. Leveau J. H. J. Gordon T. R. (2019). Persistence of *Fusarium oxysporum* f. sp. fragariae in soil through asymptomatic colonization of rotation crops. *Phytopathology* 109 770–779. 10.1094/PHYTO-11-18-0418-R 30644330

[B19] Henry P. M. Pincot D. D. A. Jenner B. N. Borrero C. Aviles M. Nam M.-H. (2021). Horizontal chromosome transfer and independent evolution drive diversification in *Fusarium oxysporum* f. sp. *fragariae*. *New Phytol.* 230 327–340. 10.1111/nph.17141 33616938 PMC7986148

[B20] Husaini A. M. Sakina A. Cambay S. R. (2018). Host–pathogen interaction in *Fusarium oxysporum* infections: where do we stand? *MPMI.* 31 889–898. 10.1094/MPMI-12-17-0302-CR 29547356

[B21] Ishikawa F. H. Souza E. A. Shoji J. Connolly L. Freitag M. Read N. D. (2012). Heterokaryon incompatibility is suppressed following conidial anastomosis tube fusion in a fungal plant pathogen. *PLoS One* 7:e31175. 10.1371/journal.pone.0031175 22319613 PMC3271119

[B22] Jelínek T. Koudela M. Kofránková V. Salava J. (2019). Effect of temperature on severity of Fusarium wilt of cabbage caused by *Fusarium oxysporum* f. sp. conglutinans. *Eur. J. Plant Pathol.* 155 1277–1286. 10.1007/s10658-019-01855-3

[B23] Kaur S. Barakat R. Kaur J. Epstein L. (2022). The effect of temperature on disease severity and growth of *Fusarium oxysporum* f. sp. apii races 2 and 4 in celery. *Phytopathology* 112 364–372. 10.1094/PHYTO-11-20-0519-R 34152209

[B24] Kurian S. Di Pietro A. Read N. (2018). Live-cell imaging of conidial anastomosis tube fusion during colony initiation in Fusarium oxysporum. *PLoS ONE* 13, 1–32. 10.1371/journal.pone.0195634 PMC5937734 29734342

[B25] Koike S. T. Gordon T. R. (2005). First report of *Fusarium* Wilt of cilantro caused by *Fusarium oxysporum* in California. *Plant Dis.* 89:1130. 10.1094/PD-89-1130A 30791290

[B26] Landa B. B. Navas-Cortés J. A. Del Mar Jiménez-Gasco M. Katan J. Retig B. Jiménez-Díaz R. M. (2006). Temperature response of chickpea cultivars to races of *Fusarium oxysporum* f. sp. *ciceris*, causal agent of *Fusarium* wilt. *Plant Dis.* 90 365–374. 10.1094/PD-90-0365 30786563

[B27] Li J. Fokkens L. van Dam P. Rep M. (2020). Related mobile pathogenicity chromosomes in *Fusarium oxysporum* determine host range on cucurbits. *Mol. Plant Pathol.* 21 761–776. 10.1111/mpp.12927 32246740 PMC7214479

[B28] Lombard L. Sandoval-Denis M. Lamprecht S. C. Crous P. W. (2019). Epitypification of *Fusarium oxysporum* – clearing the taxonomic chaos. *Persoonia* 43 1–47. 10.3767/persoonia.2019.43.01 32214496 PMC7085860

[B29] Lori G. A. Birol I. Mourelos C. A. Wolcan S. M. (2016). First report of *Fusarium oxysporum* f. sp. apii race 2 causing *Fusarium* yellows on celery in Argentina. *Plant Dis.* 100:1020.

[B30] Ma L.-J. van der Does H. C. Borkovich K. A. Coleman J. J. Daboussi M.-J. Di Pietro A. (2010). Comparative genomics reveals mobile pathogenicity chromosomes in *Fusarium*. *Nature* 464 367–373. 10.1038/nature08850 20237561 PMC3048781

[B31] Nam M. H. Kang Y. L. Kim H. G. Chun C. (2011). Infection of daughter plants by *Fusarium oxysporum* f. sp. *fragariae* through runner propagation of strawberry. *J. Hortic Sci. Technol.* 29 273–277.

[B32] Navas-Cortés J. A. Landa B. B. Méndez-Rodríguez M. A. Jiménez-Díaz R. M. (2007). Quantitative modeling of the effects of temperature and inoculum density of *Fusarium oxysporum* f. sp. *ciceris* races 0 and 5 on development of *Fusarium* wilt in chickpea cultivars. *Phytopathology* 97 564–573. 10.1094/PHYTO-97-5-0564 18943575

[B33] Nelson R. Coons G. H. Cochran L. C. (1937). *The Fusarium Yellows Disease of Celery (Apium graveolens L. var. dulce DC.). Technical Bulletin.* East Lansing: Michigan Agricultural Experiment Station, 1–74.

[B34] Oakely N. S. Hatchett B. J. McEvoy D. J. Rodrigues L. (2019). *Projected Changes in Ventura County Climate. Reno, Nevada: Western Regional Climate Center, Desert Research Institute.* Available online at: https://wrcc.dri.edu/Climate/reports.php (accessed May 29, 2022).

[B35] O’Donnell K. Al-Hatmi A. M. S. Aoki T. Brankovics B. Cano-Lira J. F. Coleman J. J. (2020). No to Neocosmospora: phylogenomic and practical reasons for continued inclusion of the *Fusarium* solani species complex in the Genus *Fusarium*. *Msphere* 5 e810–e820. 10.1128/mSphere.00810-20 32938701 PMC7494836

[B36] O’Donnell K. Gueidan C. Sink S. Johnston P. R. Crous P. W. Glenn A. (2009). A two-locus DNA sequence database for typing plant and human pathogens within the *Fusarium oxysporum* species complex. *Fungal Genet. Biol.* 46 936–948. 10.1016/j.fgb.2009.08.006 19715767

[B37] Okamoto H. Fujii S. Kato K. Yoshioka A. (1970). A new strawberry disease ‘*Fusarium* wilt.’. *Plant Protection* 24 231–235.

[B38] Orton T. J. Durgan M. E. Hulbert S. H. (1984a). Studies on the inheritance of resistance to *Fusarium oxysporum* f. sp. apii in celery. *Plant Dis.* 68 574–578.

[B39] Orton T. J. Hulbert S. H. Durgan M. E. Quiros C. F. (1984b). UC1, *Fusarium* yellows-resistant celery breeding line. *HortScience* 19:594.

[B40] Otto H. W. Paulus A. O. Snyder M. J. Endo R. M. Hart L. P. Nelson J. (1976). A crown rot of celery. *Calif. Agric.* 30 10–11. 10.1094/PD-90-0829A 30781256

[B41] Pastrana A. M. Watson D. C. Gordon T. R. (2019). Transmission of *Fusarium oxysporum* f. sp. *fragariae* through stolons in strawberry plants. *Plant Dis.* 103 1249–1251. 10.1094/PDIS-08-18-1353-RE 30932736

[B42] Paugh K. R. Gordon T. R. (2019). Effect of planting date and inoculum density on severity of *Fusarium* Wilt of lettuce in California. *Plant Dis.* 103 1498–1506. 10.1094/PDIS-09-18-1614-RE 31059386

[B43] Paugh K. R. Gordon T. R. (2020). The population of *Fusarium oxysporum* f. sp. lactucae in California and Arizona. *Plant Dis.* 104 1811–1816. 10.1094/PDIS-06-19-1228-RE 32282277

[B44] Puhalla J. E. (1984). Races of *Fusarium oxysporum* f. sp. apii in California and their genetic interrelationships. *Can. J. Bot.* 62 546–550. 10.1139/b84-080

[B45] Purugganan M. D. (2019). Evolutionary insights into the nature of plant domestication. *Curr. Biol.* 29 R705–R714. 10.1016/j.cub.2019.05.053 31336092

[B46] Retana K. Ramírez-Coché J. A. Castro O. Blanco-Meneses M. (2018). Caracterización morfológica y molecular de Fusarium oxysporum f. sp. apii asociado a la marchitez del apio en Costa Rica. *Agron. Costarricense* 42 115–126. 10.15517/rac.v42i1.32199

[B47] Rubatzky V. Quiros C. Simon P. (1999). *Carrots and Related Vegetable Umbelliferae.* New York, NY: CABI Pub.

[B48] Schneider R. W. (1984). Effects of nonpathogenic strains of *Fusarium oxysporum* on celery root infection by F. *oxysporum* f. sp. apii and a novel use of the Lineweaver-Burk double reciprocal plot technique. *Phytopathology* 74:646. 10.1094/Phyto-74-646

[B49] Scott J. C. Gordon T. R. Shaw D. V. Koike S. T. (2010). Effect of temperature on severity of *Fusarium* wilt of lettuce caused by *Fusarium oxysporum* f. sp. lactucae. *Plant Dis.* 94 13–17. 10.1094/PDIS-94-1-0013 30754388

[B50] Shahi S. Beerens B. Bosch M. Linmans J. Rep M. (2016). Nuclear dynamics and genetic rearrangement in heterokaryotic colonies of *Fusarium oxysporum*. *Fungal Genet. Biol.* 91 20–31. 10.1016/j.fgb.2016.03.003 27013267

[B51] Smith R. (2021). “Celery,” in *Carrots and Related Apiaceae Crops*, eds Geoffriau E. Simon P. W. (Wallingford: CAB International), 272–282.

[B52] Snyder W. C. Hansen H. N. (1940). The species concept in *Fusarium*. *Am. J. Bot.* 27 64–67. 10.2307/2436688

[B53] Song X. Sun P. Yuan J. Gong K. Li N. Meng F. (2021). The celery genome sequence reveals sequential paleo-polyploidizations, karyotype evolution and resistance gene reduction in Apiales. *Plant Biotechnol. J.* 19 731–744. 10.1111/pbi.13499 33095976 PMC8051603

[B54] Subbarao K. V. Elmer W. H. (2002). “Fusarium yellows of celery,” in *Compendium of Umbelliferous Crop Diseases*, eds Davis R. M. Raid R. N. (Saint Paul: American Phytopathological Society), 33–34.

[B55] van Dam P. Fokkens L. Ayukawa Y. van der Gragt M. Ter Hortst A. Brankovics B. (2017). A mobile pathogenicity chromosome in *Fusarium oxysporum* for infection of multiple cucurbit species. *Sci. Rep.* 7:9042. 10.1038/s41598-017-07995-y 28831051 PMC5567276

[B56] van Dam P. Fokkens L. Schmidt S. M. Linmans J. H. J. Kistler H. C. Ma L.-J. (2016). Effector profiles distinguish formae speciales of *Fusarium oxysporum*. *Environ. Microbiol.* 18 4087–4102. 10.1111/1462-2920.13445 27387256

[B57] van de Wouw M. Kik C. van Hintum T. van Treuren R. Visser B. (2010). Genetic erosion in crops: concept, research results and challenges. *Plant Genet. Res.* 8 1–15. 10.1017/S1479262109990062

[B58] Vangalis V. Knop M. Typas M. A. Papaioannou I. A. (2021). Establishment of conidial fusion in the asexual fungus *Verticillium dahliae* as a useful system for the study of non-sexual genetic interactions. *Curr. Genet.* 67 471–485. 10.1007/s00294-021-01157-4 33582843 PMC8139932

[B59] Vlaardingerbroek I. Beerens B. Rose L. Fokkens L. Cornelissen B. J. C. Rep M. (2016). Exchange of core chromosomes and horizontal transfer of lineage-specific chromosomes in *Fusarium oxysporum*. *Environ. Microbiol.* 18 3702–3713. 10.1111/1462-2920.13281 26941045

[B60] Yang H. Yu H. Ma L.-J. (2020). Accessory chromosomes in *Fusarium oxysporum*. *Phytopathology* 110 1488–1496. 10.1094/PHYTO-03-20-0069-IA 32692281 PMC8086798

